# Physicochemical Stability and In Vitro Antifungal Activity of *Mentha spicata* Essential Oil and *L*-Carvone Nanoemulsions Against *Stromatinia cepivora* and *Sclerotinia sclerotiorum*

**DOI:** 10.3390/molecules31203589

**Published:** 2026-10-09

**Authors:** Rodrigo Ribeiro Rocha, Lorena Alves de Melo Bessa, Geraldo Humberto Silva

**Affiliations:** Instituto de Ciências Exatas e Tecnológicas, Universidade Federal de Viçosa (UFV), Campus Rio Paranaíba, Rio Paranaíba 38810-000, MG, Brazil; rodrigor.rocha@ufv.br (R.R.R.); lorenabessa.ufv@gmail.com (L.A.d.M.B.)

**Keywords:** *Mentha spicata*, *L*-carvone, nanoemulsion, antifungal activity, phytopathogenic fungi, colloidal stability and essential oil

## Abstract

Natural bioactive compounds, such as essential oils, have attracted increasing attention for the sustainable management of plant diseases. However, their high volatility, chemical instability, and poor water solubility limit agricultural applications, highlighting the need for nanostructured delivery systems. Although *Mentha spicata* essential oil and its major constituent, *L*-carvone, exhibit well-documented antifungal activity, their nanoemulsions remain largely unexplored for controlling the soil-borne phytopathogens *Stromatinia cepivora* and *Sclerotinia sclerotiorum*. Here, oil-in-water nanoemulsions containing *M. spicata* essential oil (NEOEMS) or *L*-carvone (NEOELC) were developed and characterized regarding physicochemical properties, colloidal stability, antifungal activity, and their effects on common bean seed germination and garlic clove emergence and early growth. Both formulations remained nanometric during the 36-day storage period, despite changes in droplet size and PDI, and a limited reduction in *L*-carvone content. NEOELC completely inhibited *S. cepivora* mycelial growth at 800 µg mL^−1^, whereas NEOEMS required 1600 µg mL^−1^. Against *S. sclerotiorum*, both nanoemulsions achieved complete inhibition at concentrations of 400 µg mL^−1^, with similar IC_50_ values (162.0 and 182.8 µg mL^−1^ for NEOELC and NEOEMS, respectively). Neither formulation significantly affected bean seed germination, and garlic clove emergence was preserved; however, shoot length was lower than that of the iprodione control under field conditions. These findings support further investigation of nanoemulsions as delivery systems for natural antifungal compounds. NEOELC’s greater activity against *S. cepivora* may be related, at least in part, to its higher relative *L*-carvone content.

## 1. Introduction

White rot of *Allium* crops, such as garlic and onion, and white mold of *Phaseolus vulgaris* (common bean) are among the most destructive soil-borne fungal diseases affecting the global production of vegetables and legumes. These diseases are caused by *Stromatinia cepivora* and *Sclerotinia sclerotiorum*, respectively [1,2]. Both pathogens exhibit remarkable persistence due to the long-term survival of their sclerotia in soil, where they may remain viable for several years, posing a major challenge for disease management and highlighting the need for innovative crop protection strategies. *S. cepivora* can cause severe yield losses, frequently leading to complete crop failure in *Allium* cultivation and substantially increasing production costs [2,3,4]. Likewise, *S. sclerotiorum* induces stem rot and other destructive symptoms in common bean, resulting in significant economic losses [5].

Chemical control of these soil-borne diseases remains challenging. Although synthetic fungicides continue to play an essential role in disease management, their effectiveness is often limited by the need for repeated applications. Furthermore, their intensive use is associated with significant environmental concerns, including soil and water contamination through leaching, toxicity to non-target organisms, and the emergence of fungicide-resistant pathogen populations [5,6,7,8,9,10]. Consequently, alternative approaches based on bioactive natural compounds, host genetic resistance, and biological control have attracted increasing attention as sustainable disease management strategies [4,5,11].

Among these alternatives, essential oils (EOs), which are composed predominantly of terpenoids and phenylpropanoids, have emerged as promising natural antifungal agents. Their biological activity is strongly influenced by their molecular structure, volatility, lipophilicity, and chemical reactivity. Several studies have demonstrated the antifungal activity of EOs against a wide range of phytopathogens, including species of *Sclerotinia* and *Stromatinia* [7,12,13,14,15,16,17,18,19].

The essential oil of *M. spicata* (spearmint) is rich in oxygenated monoterpenes, with *L*-carvone representing its major constituent. The α, β-unsaturated ketone structure of *L*-carvone has been associated with membrane-disrupting properties and broad-spectrum antimicrobial activity [20,21,22]. This monoterpene compromises membrane integrity, disrupts ergosterol biosynthesis, and interferes with fungal respiration in several agriculturally important phytopathogens, including species of *Colletotrichum*, *Fusarium*, and *Aspergillus* [15,17,23]. Despite the well-documented antifungal properties of *L*-carvone, studies investigating its nanoemulsion formulations for the control of economically important soil-borne phytopathogens remain scarce. In addition to their biological activity, terpenes are renewable natural products with a favorable environmental profile, making them attractive candidates for sustainable crop protection [6,7,10,15,19]. Nevertheless, the direct application of essential oils is hindered by several intrinsic limitations, including high vapor pressure, oxidative instability, volatility, photodegradation, and poor water solubility, all of which compromise their bioavailability and field performance [7,24,25].

Nanoemulsion-based formulations have been investigated as delivery systems for essential oils and other hydrophobic bioactive compounds. Previous studies have reported that their nanometric droplet size and high interfacial area may favor aqueous dispersion and influence the physicochemical behavior and biological performance of incorporated compounds [24,26,27,28,29,30,31,32]. However, these effects depend on formulation composition, active compound, and experimental conditions and should be demonstrated by direct comparison with the corresponding non-formulated compounds.

Despite significant advances in nanoemulsion technology, limited information is available regarding the formulation, physicochemical stability, and antifungal performance of nanoemulsified *M. spicata* essential oil and *L*-carvone against economically important soil-borne phytopathogens. We hypothesized that nanoemulsification of *M. spicata* essential oil (NEOEMS) and *L*-carvone (NEOELC) would produce physicochemically stable colloidal systems with antifungal activity against soil-borne phytopathogens and acceptable compatibility with the evaluated plant materials.

Therefore, the objectives of the present study were to (i) formulate and physicochemically characterize oil-in-water nanoemulsions containing *M. spicata* essential oil or *L*-carvone; (ii) evaluate their colloidal stability and *L*-carvone retention during storage; and (iii) determine their antifungal activity against *S. cepivora* and *S. sclerotiorum* and evaluate their effects on common bean seed germination and seedling development and on garlic clove emergence and early growth under in vitro and field conditions.

## 2. Results

### 2.1. Chemical Composition of Mentha spicata Essential Oil

**Figure 1:**
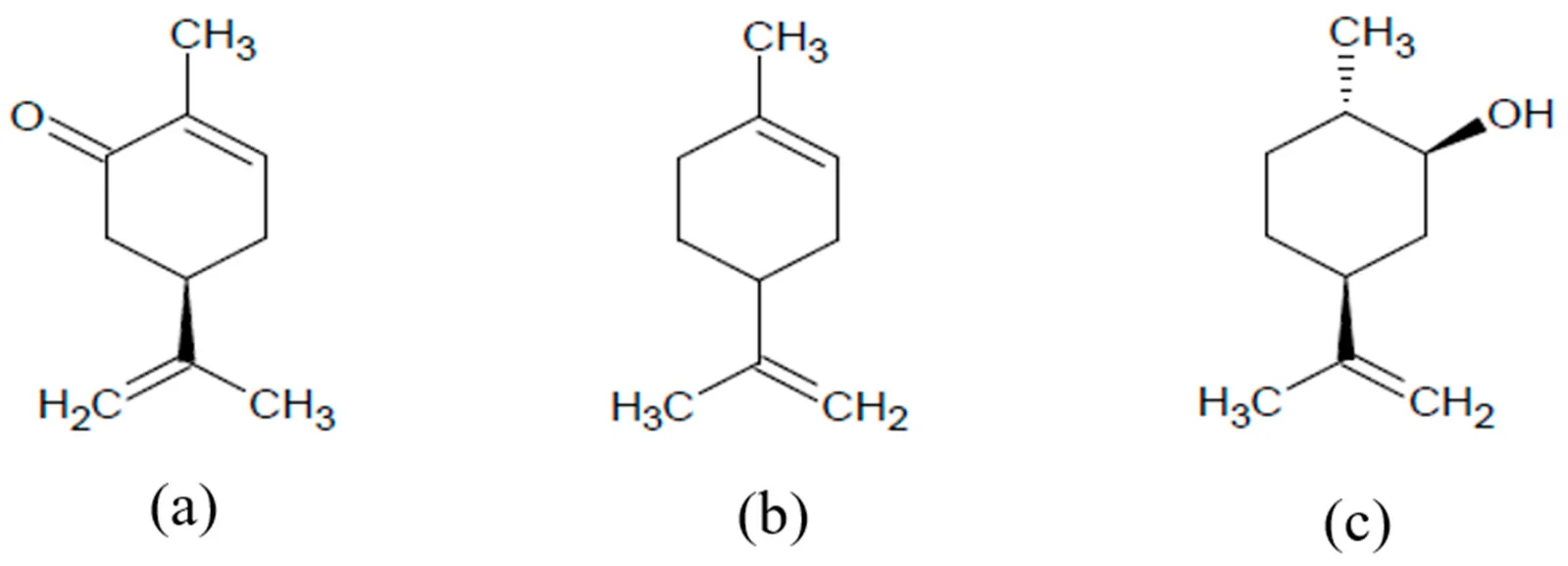
Chemical structures of the major constituents of *Mentha spicata* essential oil: (**a**) *L*-carvone, (**b**) limonene, and (**c**) dihydrocarveol. Structures were generated using ACD/ChemSketch (2015).

GC–MS analysis of the *M. spicata* essential oil enabled the tentative annotation of 16 of the 17 detected compounds based on mass spectral library matching and arithmetic retention indices. Based on relative chromatographic peak areas (Table 1), the major constituents were *L*-carvone (73.35%), limonene (11.21%), and dihydrocarveol (3.34%), which together accounted for 87.90% of the total essential oil composition (Figure 1).

The major compounds exhibited spectral similarity indices of 95% or higher relative to the reference mass spectral library used for compound annotation.

### 2.2. L-Carvone Quantification and Chemical Stability in the Nanoemulsions

**Figure 2:**
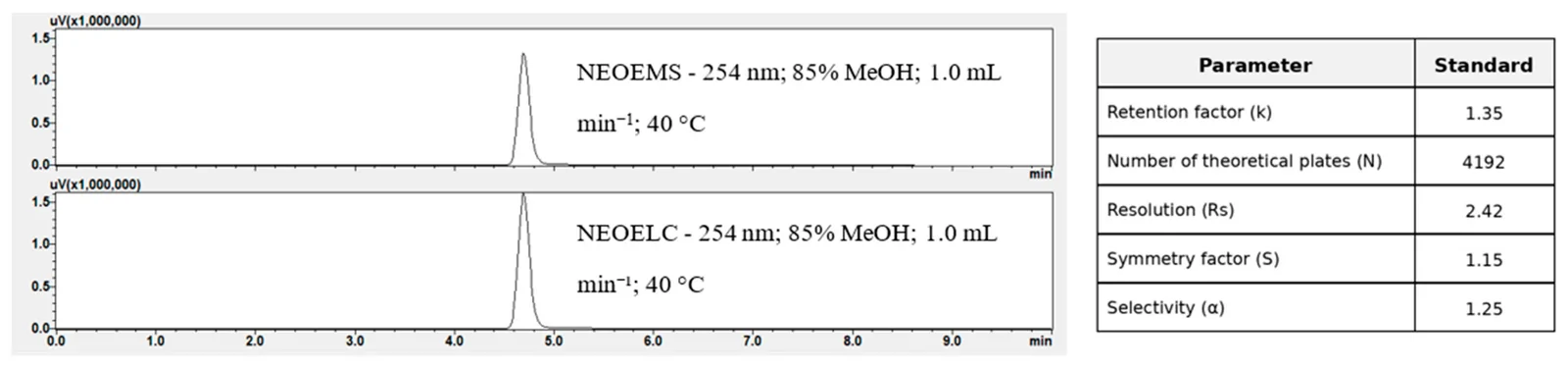
Representative HPLC–UV chromatograms of NEOEMS and NEOELC obtained during the initial development of the chromatographic method using the Shimadzu LC-20A system at 254 nm. The inset summarizes the system-suitability parameters obtained for the *L*-carvone standard.

The chromatographic separation of *L*-carvone was initially established using the Shimadzu LC-20A system with UV detection at 254 nm. Representative chromatograms obtained during this initial method-development stage showed adequate separation of *L*-carvone from the formulation components (Figure 2). After transferring the method to the Agilent 1260 HPLC–DAD system, we evaluated the analytical response of *L*-carvone at different wavelengths and selected 270 nm for validation and quantitative analysis. Comparison of the chromatographic profiles of the *L*-carvone analytical standard, placebo formulation, NEOELC, and NEOEMS showed adequate chromatographic selectivity for *L*-carvone under the analytical conditions employed.

Linearity was evaluated over the concentration range of 0.05–1.00 mg mL^−1^. The calibration curve prepared with *L*-carvone standard in methanol followed y = 4210.11x + 19.17, with a coefficient of determination (R^2^) of 0.9994. No significant lack of fit was observed (*p* = 0.104), supporting the adequacy of the linear regression model within the investigated concentration range.

A possible matrix effect was evaluated by comparing the calibration curve prepared in methanol with a calibration curve prepared in the presence of the placebo formulation. The latter followed y = 4221.26x + 47.81 (R^2^ = 0.9976), with no significant lack of fit (*p* = 0.206). The slopes of the calibration curves obtained in solvent and in the presence of placebo differed by only 0.26%. They were not significantly different (*p* = 0.848), indicating that the formulation matrix did not significantly affect the chromatographic response of *L*-carvone under the analytical conditions employed.

Based on the calibration data, the LOD and LOQ were estimated at 0.0293 and 0.0888 mg mL^−1^, respectively. Accuracy was further evaluated by recovery experiments in the placebo formulation at 80, 100, and 120% of the nominal *L*-carvone concentration. Mean recoveries were 96.30 ± 1.07%, 101.05 ± 0.71%, and 98.93 ± 3.51%, respectively, resulting in an overall mean recovery of 98.76 ± 2.78%.

Repeatability was evaluated using six independently prepared NEOELC samples, each analyzed in duplicate. The mean *L*-carvone concentration determined for these independent preparations was 45.37 ± 1.52 mg mL^−1^, with an RSD of 3.34%, demonstrating adequate intra-day repeatability of the analytical procedure. The analytical validation results are summarized in Table 2.

Direct HPLC–DAD analysis of the *M. spicata* essential oil used to prepare NEOEMS showed an *L*-carvone content of 67.43 ± 0.47% (*w*/*w*), with an RSD of 0.70% (*n* = 3).

### 2.3. Macroscopic Evaluation and Physical Stability of NEOEMS and NEOELC

Using the standardized preparation conditions described in item 4.3, immediately after preparation, both NEOEMS and NEOELC formed slightly opalescent, homogeneous, and monophasic dispersions, with no visible signs of flocculation, sedimentation, creaming, or phase separation. These macroscopic characteristics were observed across the independently prepared replicates, providing an initial indication that the dispersed systems formed.

Following centrifugation at 1000 and 2500 rpm, no sedimentation, creaming, or phase separation was observed in any replicate. At 3500 rpm, one NEOELC replicate exhibited sediment formation, whereas the remaining replicates showed no visible phase separation. During the temperature-cycling test, all replicates remained monophasic with no visible changes following exposure to 4 °C. In contrast, exposure to 45 °C resulted in sedimentation and/or phase separation in most replicates, although one replicate of each formulation remained visually unchanged. These observations indicate greater susceptibility of both formulations to physical destabilization at the higher temperature.

### 2.4. Physicochemical Properties (Dynamic Light Scattering and Zeta Potential) and L-Carvone Stability

The physicochemical properties of the NEOEMS and NEOELC nanoemulsions were monitored over 36 days of storage (Table 3). Both formulations maintained mean droplet diameters within the nanometric range throughout the entire period, although an increase in droplet size was observed. The mean droplet diameter of NEOELC increased from 80.84 to 186.47 nm, whereas that of NEOEMS increased from 119.66 to 152.83 nm. In parallel, the PDI decreased from 0.39 to 0.20 for NEOELC and from 0.35 to 0.27 for NEOEMS, indicating a narrower droplet-size distribution at the end of storage. Thus, despite measurable changes in their colloidal properties over time, both formulations maintained mean droplet diameters within the nanometric range during the evaluated period.

The zeta potential showed only modest variation during storage, changing from −15.26 to −14.39 mV for NEOELC and from −14.64 to −17.17 mV for NEOEMS. These values were below the ±30 mV range commonly associated with strong electrostatic stabilization.

Electrical conductivity remained low throughout the study. NEOEMS exhibited little variation during storage, whereas NEOELC increased from 0.02 to 0.12 mS cm^−1^.

The pH remained close to neutrality, ranging from 6.5 to 7.3, with only minor fluctuations throughout the storage period.

In addition to colloidal properties, the chemical stability of *L*-carvone was evaluated during storage (Table 3). In NEOELC, the *L*-carvone concentration decreased moderately from 42.86 ± 0.31 mg mL^−1^ on Day 1 to 41.23 ± 2.32 mg mL^−1^ on Day 36, corresponding to a retention of approximately 96.2% of the initial concentration. In NEOEMS, the *L*-carvone concentration decreased from 27.68 ± 0.36 mg mL^−1^ on Day 1 to 26.00 ± 0.10 mg mL^−1^ on Day 36, corresponding to a retention of approximately 93.9%. These results indicate substantial preservation of the *L*-carvone content in both formulations during storage. Chemical stability was evaluated independently of colloidal stability, which was assessed based on changes in droplet size, PDI, zeta potential, and other physicochemical parameters.

### 2.5. Antifungal Activity of NEOEMS and NEOELC Against S. cepivora and S. sclerotiorum

**Figure 3:**
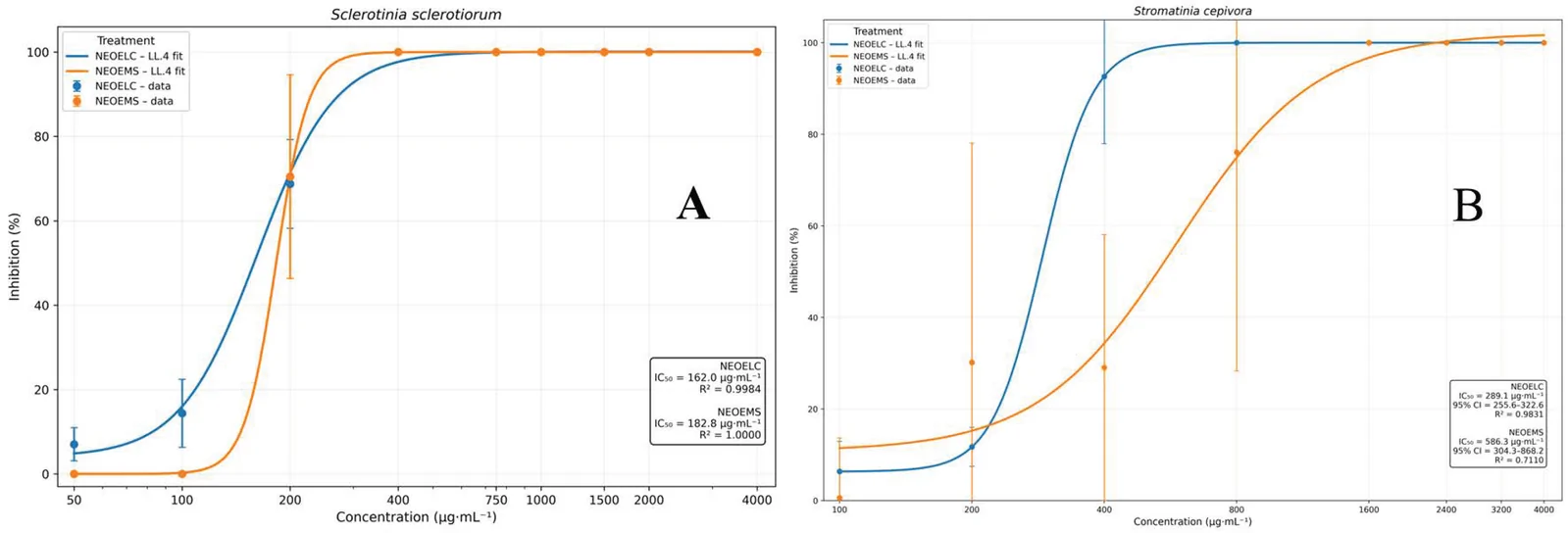
Dose–response curves of NEOEMS and NEOELC against (**A**) *Sclerotinia sclerotiorum* and (**B**) *Stromatinia cepivora*. Points and error bars represent the mean ± SD of four independent plates per concentration. Solid lines represent the four-parameter log-logistic models fitted to the individual plate values.

NEOELC and NEOEMS exhibited concentration-dependent antifungal activity against both phytopathogenic fungi, with the magnitude of the response depending on the formulation and the fungal species (Table 4). The corresponding dose–response curves obtained from the four-parameter log-logistic models are shown in Figure 3.

The control treatments showed the expected contrasting responses for both phytopathogens. The distilled-water and blank nanoemulsion controls showed no inhibition of fungal growth (0% inhibition), whereas iprodione markedly reduced fungal growth, although complete inhibition was not observed in all replicates. These results indicate that the blank formulation did not exhibit detectable antifungal activity under the experimental conditions.

Against *S. cepivora*, NEOELC showed markedly greater antifungal potency than NEOEMS, with an IC_50_ of 289.1 µg mL^−1^ and an MIC of 800 µg mL^−1^, compared with 586.3 µg mL^−1^ and 1600 µg mL^−1^, respectively, for NEOEMS. Thus, NEOELC required approximately half the NEOEMS concentration to achieve both 50% inhibition and complete growth inhibition. This difference is also evident in the dose–response profiles (Figure 3B), in which the NEOELC curve is shifted toward lower concentrations relative to NEOEMS.

Against *S. sclerotiorum*, both formulations showed greater antifungal activity than that observed against *S. cepivora*. Complete inhibition was achieved at 400 µg mL^−1^ for both formulations, while the estimated IC_50_ values were 162.0 µg mL^−1^ for NEOELC and 182.8 µg mL^−1^ for NEOEMS. The proximity of the dose–response curves and their IC_50_ values (Figure 3A) indicates comparable antifungal potency against *S. sclerotiorum*.

Overall, the results indicate that the influence of formulation composition was pathogen-dependent: the difference between NEOELC and NEOEMS was pronounced against *S. cepivora*, whereas both formulations exhibited comparable activity against *S. sclerotiorum*.

### 2.6. Effects of NEOEMS and NEOELC on Common Bean Seed Germination and Garlic Clove Emergence and Early Growth

The effects of NEOEMS and NEOELC applied at different concentrations were evaluated with respect to seed germination and early seedling development (Table 5). The treatments consisted of three application rates of each nanoemulsion, in addition to Tween 80 and distilled water controls.

Seed germination remained high across all treatments, ranging from 96% to 99%, with no statistically significant differences among nanoemulsion formulations, application rates, or controls (*p* > 0.05). These findings indicate that the evaluated formulations did not produce a statistically detectable reduction in germination under the experimental conditions evaluated, even at the highest concentrations tested during the initial stage of seed development.

Leaf fresh weight did not differ significantly among treatments, indicating that early shoot growth was not adversely affected. Although leaf dry weight was significantly influenced by treatment, the highest values were observed in the Tween 80 control, whereas most nanoemulsion treatments exhibited intermediate values without a clear dose–response relationship. These results suggest that the observed variation did not provide evidence of a consistent concentration-dependent effect of the nanoemulsions.

Root-related parameters, including root fresh weight, root dry weight, and root length, were not significantly affected by any treatment (*p* > 0.05).

Garlic clove emergence reached 100% in all treatments, with emergence defined as the appearance of roots and/or shoots. Therefore, neither NEOELC nor NEOEMS produced a statistically detectable reduction in the resumption of vegetative growth of garlic cloves under the experimental conditions evaluated.

Early seedling growth was evaluated by measuring shoot length (SL) and root length (RL) (Table 6). For shoot length, a progressive increase in growth was observed with increasing nanoemulsion concentrations up to 1500 µg mL^−1^ for both formulations. In the NEOELC treatment, shoot length increased from 10.95 cm at 250 µg mL^−1^ to 30.70 cm at 1500 µg mL^−1^. Similarly, NEOEMS promoted an increase from 15.87 cm to 30.26 cm over the same concentration range. At 1500 µg mL^−1^, both formulations produced significantly greater shoot growth than the Tween 80 control, indicating enhanced aerial development under these conditions.

No significant differences in root length (RL) were observed among concentrations within either nanoemulsion. The only significant difference between formulations was detected at 750 µg mL^−1^, where NEOEMS produced greater root length than NEOELC. None of the treatments differed significantly from the distilled water or iprodione controls under the experimental conditions evaluated.

In the field experiment conducted using a randomized complete block design (RCBD), plant emergence did not differ significantly among treatments (*p* > 0.05), ranging from 85.83% to 92.50% (Table 7). These findings indicate that neither NEOEMS nor NEOELC produced a statistically significant reduction in plant emergence under the conditions evaluated.

In contrast, the treatments had a significant effect on shoot length (SL) (*p* = 0.00499). The iprodione control exhibited the greatest shoot length, with a mean value of 10.37 cm, whereas the NEOEMS (2000 µg mL^−1^) and NEOELC (1500 µg mL^−1^) treatments resulted in lower mean shoot lengths of 8.03 and 7.46 cm, respectively. Therefore, although the nanoemulsions did not impair plant emergence, they were associated with reduced early shoot growth compared with the iprodione control.

## 3. Discussion

The predominance of *L*-carvone in the essential oil is particularly relevant, considering its previously reported antifungal activity against several phytopathogenic fungi, including *Colletotrichum musae*, *Colletotrichum gloeosporioides*, and *Fusarium subglutinans* f. sp. ananas [34]. Limonene, the second most abundant constituent, has also been reported to have antimicrobial activity, further supporting the essential oil’s biological potential [35].

The results show that *M. spicata* essential oil and *L*-carvone were successfully incorporated into oil-in-water nanoemulsions that retained nanometric droplet sizes during the evaluated storage period and exhibited antifungal activity against the two soil-borne phytopathogens investigated. Antifungal activity differed between formulations and depended on the fungal species. The physicochemical characterization also revealed measurable changes in droplet size and PDI during storage, indicating that formulation performance should be interpreted by considering colloidal and chemical stability separately from biological activity.

The physicochemical characterization confirmed the formation of nanoemulsions with nanometric droplet sizes and PDI values ranging from 0.20 to 0.39 during the evaluated storage period. These PDI values indicate that the droplet-size distributions varied from moderately polydisperse to relatively narrower distributions, rather than being uniformly characterized by low polydispersity. These findings are consistent with previous studies reporting the successful use of nonionic Tween-type surfactants, particularly Tween 80, for encapsulating essential oils and improving their dispersion in aqueous media [24,31]. The smaller initial droplet diameter observed for NEOELC compared with NEOEMS may be related to the lower chemical complexity of pure *L*-carvone relative to the multicomponent composition of the essential oil, which may facilitate emulsification and reduce interfacial heterogeneity, and thereby contribute to smaller droplets [28,29,31].

During storage, both formulations exhibited an increase in mean droplet diameter while remaining within the nanometric range, showing that the formulations retained their nanometric character despite measurable changes in their colloidal properties over time. The observed increase in droplet size may be associated with destabilization processes such as Ostwald ripening and/or incipient coalescence. In Ostwald ripening, molecules are transferred from smaller to larger droplets through the continuous phase, whereas coalescence involves the merging of droplets. Because these mechanisms were not directly investigated in the present study, the observed increase in droplet size cannot be attributed to a specific destabilization pathway.

Although the absolute zeta potential values remained below the conventional ±30 mV threshold generally associated with electrostatic stabilization, the persistence of the dispersions without macroscopic phase separation during the evaluated storage period suggests that electrostatic repulsion was not the sole mechanism contributing to colloidal stabilization. As Tween 80 is a nonionic surfactant, steric repulsion provided by its polyoxyethylene chains is expected to contribute to minimizing droplet aggregation and coalescence [29,31]. This behavior is characteristic of nanoemulsions stabilized with nonionic surfactants and supports the interpretation that zeta potential alone is not a sufficient predictor of the stability of such systems.

The antifungal activity of NEOELC and NEOEMS demonstrated that *L*-carvone and *M. spicata* essential oil remained biologically active when incorporated into the nanoemulsion systems. Antifungal activity has also been reported for nanoemulsion-based systems containing other essential oils, although differences in the active phase, carrier composition, and fungal targets preclude direct quantitative comparisons [36]. A key practical feature of these oil-in-water formulations is their ability to disperse hydrophobic bioactive compounds in an aqueous continuous phase, which may facilitate dilution and handling and support future adaptation to conventional agricultural spraying systems. Previous studies have reported that nanoemulsion systems may improve the dispersion and distribution of hydrophobic bioactive compounds in aqueous environments [37,38]. The study did not include matched treatments containing non-formulated *L*-carvone or *M. spicata* essential oil because their low water solubility would require additional solvents or dispersants and the corresponding vehicle controls. Therefore, the present results demonstrate the antifungal activity of the developed formulations but do not establish whether nanoemulsification increased efficacy relative to the corresponding free active compounds. Such comparisons will require appropriately matched solvent or dispersant controls in future studies.

From a mechanistic perspective, essential oil nanoemulsions may exert antifungal activity through multiple pathways, including increased membrane permeability, disruption of fungal membrane integrity, and interference with essential cellular processes such as ergosterol biosynthesis, ultimately leading to loss of cellular integrity and pathogen death. Recent evidence indicates that carvone may inhibit ergosterol biosynthesis and damage fungal mitochondria, contributing to its antifungal activity [23].

In the present study, NEOELC exhibited greater antifungal potency against *S. cepivora*, with an MIC of 800 μg mL^−1^ and an IC_50_ of 289.1 μg mL^−1^, whereas NEOEMS presented an MIC of 1600 μg mL^−1^ and an IC_50_ of 586.3 µg mL^−1^. The greater activity of NEOELC may be related to its higher relative *L*-carvone content; however, the present design does not allow the contribution of *L*-carvone to be separated from other formulation-related effects. In contrast, both formulations exhibited high activity against *S. sclerotiorum*, with identical MIC values of 400 μg mL^−1^ and similar IC_50_ values, indicating comparable susceptibility of this fungus to both nanoemulsions. Previous studies have likewise demonstrated the antifungal activity of *L*-carvone against several phytopathogens, including *Fusarium pallidoroseum* and *Fusarium solani* [39] and *Colletotrichum* spp. [40]. The greater antifungal activity of NEOELC than NEOEMS against *S. cepivora* may be related to the higher proportion of *L*-carvone in its active phase. While the active phase of NEOELC consisted almost entirely of *L*-carvone, HPLC–DAD analysis showed that *L*-carvone represented approximately 67.4% of the *M. spicata* essential oil used in NEOEMS. Therefore, at equivalent formulation concentrations, NEOELC provided a higher relative amount of *L*-carvone than NEOEMS. This difference may have contributed to the lower IC_50_ and MIC values observed for NEOELC. Nevertheless, the contribution of other essential oil constituents, as well as synergistic or antagonistic interactions among them, cannot be excluded.

Neither NEOEMS nor NEOELC significantly affected common bean seed germination, and no substantial changes were observed in fresh biomass, dry biomass, or root length at the concentrations evaluated. Similarly, in the in vitro garlic assay, emergence reached 100% in all treatments, and root growth did not differ significantly from the distilled water or iprodione controls.

Under field conditions, although plant emergence was not impaired, shoot length was significantly lower in garlic plants treated with NEOEMS and NEOELC than in those treated with iprodione. These results indicate that preservation of germination or emergence does not necessarily imply an absence of effects on subsequent vegetative growth. Therefore, the formulations should not be considered non-phytotoxic based solely on the present assays, and further dose optimization and crop-safety evaluation are warranted.

This study also has limitations. The mechanisms of fungal inhibition were inferred from the literature rather than experimentally confirmed, and long-term evaluations under open-field conditions were not performed. Future studies should address formulation persistence, long-term stability, crop compatibility, and antifungal efficacy under different environmental conditions.

Overall, the nanoemulsions containing *L*-carvone or *M. spicata* essential oil showed antifungal activity while preserving germination and emergence under the tested conditions. However, the observed reduction in garlic shoot growth under field conditions highlights the need for additional studies on crop safety and field performance before practical application.

## 4. Materials and Methods

### 4.1. Reagents

*M. spicata* essential oil was purchased from Ferquima Ind. & Com. Ltda. (Vargem Grande Paulista, Brazil). *L*-carvone (purity ≥ 98%) and polyoxyethylene sorbitan monooleate (Tween 80) were obtained from Sigma-Aldrich (St. Louis, MO, USA). Potato dextrose agar (PDA) was purchased from Kasvi (São José dos Pinhais, Brazil). All reagents were of analytical grade and used without further purification. *S. cepivora* and *S. sclerotiorum* isolates were maintained on PDA before the antifungal assays in a biochemical oxygen demand (BOD) incubator at 17 °C.

### 4.2. Chemical Characterization of the Essential Oil

The chemical composition of the essential oil (EO) was determined by gas chromatography–mass spectrometry (GC–MS) using Shimadzu QP2010 system (Shimadzu, Kyoto, Japan) equipped with an RTX-5MS capillary column (30 m × 0.25 mm × 0.25 μm film thickness; Restek Corporation, Bellefonte, PA, USA). Helium was used as the carrier gas at a flow rate of 1.0 mL min^−1^. A 1 μL aliquot of the sample was injected in split mode (20:1), with the injector temperature set at 220 °C. The oven temperature program consisted of an initial ramp from 60 to 200 °C at 2 °C min^−1^, followed by a second ramp from 200 to 250 °C at 5 °C min^−1^. The mass spectrometer operated under electron ionization (EI) at 70 eV, with a scan range of *m*/*z* 29–450. Compounds were tentatively annotated based on two complementary criteria: comparison of the experimental mass spectra with those available in the mass spectral library and comparison of the calculated arithmetic retention indices (AI) with reference values reported by Adams [33]. Because authentic reference standards were not analyzed under the same GC–MS conditions, the compound assignments were considered tentative rather than definitively confirmed. Peaks for which these criteria did not allow a reliable annotation were reported as unidentified (NI).

### 4.3. Preparation of Nanoemulsions (NEOEMS and NEOELC)

Both formulations consisted of 4.35% (*w*/*w*) oil phase, 8.70% (*w*/*w*) Tween 80, and 86.95% (*w*/*w*) distilled water, with a total mass of 34.5 g. All components were weighed sequentially directly into the same Erlenmeyer flask, first the oil phase, followed by Tween 80 and finally distilled water, to minimize material losses during preparation. Each formulation was prepared independently in triplicate in a 150 mL Erlenmeyer flask (45 mm inner diameter, 95 mm height). The mixtures were homogenized using an Ultra-Turrax high-shear homogenizer (IKA T18; IKA-Werke GmbH & Co. KG, Staufen, Germany), with the dispersing element positioned centrally in the flask, approximately 5 mm above the bottom. Homogenization was performed at 15,000 rpm for 5 min at room temperature. The resulting nanoemulsions were transferred to amber glass vials and protected from light until analysis.

In biological assays in which treatments were expressed in µg mL^−1^, NEOELC and NEOEMS concentrations refer to *L*-carvone and *M. spicata* essential oil, respectively, delivered through the corresponding nanoemulsions. These concentrations were calculated from the active-phase content of each formulation and the dilution used to prepare the culture medium or treatment solution, as applicable.

### 4.4. Physicochemical Characterization and Stability of the Nanoemulsions (NEs)

The nanoemulsions (NEs) were characterized in terms of mean hydrodynamic droplet size, polydispersity index (PDI), zeta potential (ζ), pH, and electrical conductivity. Their stability was monitored for 36 days by visual inspection and periodic evaluation of these physicochemical parameters. Macroscopic stability was assessed by visual inspection for phase separation, precipitation, sedimentation, creaming, color changes, and other visible alterations. For the centrifugation test, the samples were sequentially centrifuged at 1000, 2500, and 3500 rpm for 15 min at each speed and visually inspected after each centrifugation step. For the temperature-cycling test, the nanoemulsions were subjected to six consecutive cycles between 4 and 45 °C, with samples maintained for 24 h at each temperature (48 h per cycle; 12 days in total). The formulations were evaluated before the test and after completion of the sixth cycle.

The mean hydrodynamic droplet size, polydispersity index (PDI), and zeta potential (ζ) of the nanoemulsions were determined using a Zetasizer Pro Blue (Blue Series, model 3200HAS; Malvern Panalytical, Malvern, UK). Measurements were performed at 25 °C after diluting the samples 1:10 (*v*/*v*) with deionized water immediately before analysis. The 1:10 dilution was used to minimize multiple scattering and obtain an optical count rate suitable for the instrument. Because dilution may alter the properties of the continuous phase and interparticle interactions, all formulations were analyzed under the same standardized dilution conditions. Therefore, the results were interpreted comparatively and represent the diluted dispersions rather than the undiluted formulations. The hydrodynamic diameter was determined by dynamic light scattering (DLS) and expressed as the Z-average diameter.

Droplet size distributions were also evaluated on an intensity-weighted basis. All DLS measurements were performed at a backscatter detection angle of 173°. Zeta potential was determined from electrophoretic mobility by laser Doppler microelectrophoresis, and ζ values were calculated according to the Smoluchowski model. The instrument software was configured using water properties as the dispersant (refractive index = 1.33; viscosity = 1.0 cP). A folded capillary cell (DTS1070, Malvern Panalytical, Malvern, UK) was used for zeta potential measurements, whereas DLS measurements were performed using a quartz cuvette. Results are expressed as the mean ± standard deviation (SD) of three independent determinations (*n* = 3) for each formulation.

The formulations were further characterized by measuring pH and electrical conductivity using a benchtop pH meter and conductivity meter (Quimis, Diadema, SP, Brazil) at room temperature.

### 4.5. Quantification of L-Carvone in the Essential Oil and Nanoemulsions and Evaluation of Chemical Stability

The chromatographic conditions for *L*-carvone analysis were initially developed using a Shimadzu LC-20A Prominence high-performance liquid chromatograph equipped with a UV–Vis detector (Shimadzu, Kyoto, Japan). Separation was performed on a reversed-phase C18 column (Luna, Phenomenex, Torrance, CA, USA, 250 × 4.6 mm, 5 μm) under isocratic conditions. The mobile phase consisted of methanol and 0.1% (*v*/*v*) aqueous formic acid (85:15, *v*/*v*), delivered at a constant flow rate of 1.0 mL min^−1^. During this initial method-development stage, detection was performed at 254 nm.

For analytical validation and quantitative determination, the chromatographic procedure was subsequently transferred to an Agilent 1260 HPLC system (Agilent Technologies, Santa Clara, CA, USA) equipped with a G7111B 1260 Quaternary Pump, a G7129A 1260 Vialsampler autosampler, and a G7117C 1260 DAD HS diode-array detector. The same reversed-phase C18 column and mobile-phase composition established during initial method development were maintained. The diode-array detector was used to evaluate the analytical response of *L*-carvone at different wavelengths, and 270 nm was selected for quantitative analysis because it provided the most suitable response under the chromatographic conditions used. The column temperature was maintained at 40 °C, the injection volume was 10 μL, and the mobile-phase flow rate was 1.0 mL min^−1^.

For quantitative analysis, 100 μL of each nanoemulsion was transferred to a 10 mL volumetric flask and diluted to volume with methanol, corresponding to a 100-fold dilution factor. This procedure disrupted the colloidal system and solubilized *L*-carvone prior to chromatographic analysis. Quantification was based on the chromatographic peak area of *L*-carvone, and concentrations determined in the diluted samples were corrected by the dilution factor and expressed as mg mL^−1^ of the original nanoemulsion.

The analytical procedure was evaluated for selectivity, linearity, matrix effect, limits of detection and quantification (LOD and LOQ), accuracy, and repeatability. Selectivity was assessed by comparing chromatographic profiles obtained for the *L*-carvone analytical standard, placebo formulation, NEOELC, and NEOEMS. The placebo formulation contained Tween 80 and water but no *L*-carvone or *M. spicata* essential oil.

Linearity was evaluated over the concentration range of 0.05–1.00 mg mL^−1^ using six concentration levels (0.05, 0.10, 0.25, 0.50, 0.75, and 1.00 mg mL^−1^), with each concentration independently analyzed in triplicate. Calibration curves were constructed by linear regression of chromatographic peak area versus *L*-carvone concentration. The adequacy of the linear regression model was assessed using the coefficient of determination (R^2^) and the lack-of-fit test.

The matrix effect was evaluated by constructing two calibration curves over the same concentration range: one containing *L*-carvone analytical standard in solvent and another containing *L*-carvone in the presence of the placebo formulation. The slopes of the two regression models were statistically compared to determine whether components of the nanoemulsion matrix significantly affected *L*-carvone’s chromatographic response.

The LOD and LOQ were estimated from the variability of the analytical response and the slope of the calibration curve.

The accuracy of the analytical method was assessed through recovery experiments using a placebo formulation that was spiked with specific amounts of *L*-carvone. This was done at three levels corresponding to 80%, 100%, and 120% of the nominal *L*-carvone concentration. Three independently prepared samples were analyzed at each concentration level, and the recovery was calculated as the percentage of the added *L*-carvone that was successfully recovered by the analytical procedure.

Repeatability (intra-day precision) was evaluated using six independently prepared NEOELC samples at the nominal formulation concentration. Each independent preparation was analyzed in duplicate under the same chromatographic conditions on the same day. Repeatability was expressed as the relative standard deviation (RSD) of the *L*-carvone concentrations determined for the six independent preparations.

The *L*-carvone content in *M. spicata* essential oil, NEOELC, and NEOEMS was quantified using the HPLC-DAD procedure described above. For analysis of the essential oil, a 0.5 mg mL^−1^ solution was prepared by accurately weighing 50 mg of *M. spicata* essential oil and diluting to 100 mL with methanol. The *L*-carvone content in NEOELC and NEOEMS was determined on day 1 and after 36 days of storage.

### 4.6. Evaluation of the Antifungal Activity of NEOEMS and NEOELC

The antifungal activity assays were conducted at the Laboratory for the Development of Natural Agrochemicals, Federal University of Viçosa, Rio Paranaíba Campus (UFV-CRP). The *S. cepivora* and *S. sclerotiorum* isolates used in this study were cultured from viable sclerotia on potato dextrose agar (PDA) plates.

Potato dextrose agar (PDA) medium was prepared at a concentration of 39 g L^−1^ in distilled water and sterilized in a vertical autoclave at 121 °C for 15 min. After sterilization, the medium was transferred to a laminar flow hood. The in vitro antifungal activity was evaluated using the poisoned food technique, in which the treatments were incorporated directly into the culture medium. This method is widely employed to assess the inhibition of mycelial growth in phytopathogenic fungi [41,42].

The complete nanoemulsion formulations were incorporated into molten PDA, and the reported concentrations refer to the final concentrations of their respective active phases in the culture medium. Thus, NEOELC concentrations were expressed as *L*-carvone equivalents, whereas NEOEMS concentrations were expressed as *M. spicata* essential oil equivalents. The concentrations were calculated considering the active-phase content of each formulation and its dilution in the final volume of PDA. For *S. cepivora*, the final active-phase concentrations evaluated were 100, 200, 400, 800, 1600, and 4000 μg mL^−1^. For *S. sclerotiorum*, an additional concentration of 50 μg mL^−1^ was included, resulting in concentrations of 50, 100, 200, 400, 800, 1600, and 4000 μg mL^−1^. Iprodione at 100 mg L^−1^ was used as the positive control, whereas PDA alone and a blank nanoemulsion containing Tween 80 and water were used as negative controls.

After thorough homogenization, the culture medium for each treatment was poured into four independent Petri dishes (*n* = 4 per concentration), with each plate considered an independent experimental unit. The plates were inoculated with mycelial plugs obtained from the actively growing margins of 7-day-old cultures. After inoculation, the plates were sealed with plastic film and incubated at 25 °C. For each fungal species, growth was assessed when the colony in the corresponding untreated control reached the edge of the Petri dish. Fungal growth was determined by measuring colony diameter for *S. sclerotiorum* and digitally quantifying colony area (cm^2^) for *S. cepivora*. Each plate provided one independent response value for the dose–response analysis.

The percentage of mycelial growth inhibition (MGI) was calculated according to the following equation:

MGI (%) = [(Dc − Dt)/Dc] × 100

The minimum inhibitory concentration (MIC) was defined as the lowest tested concentration resulting in complete inhibition of fungal growth. The half-maximal inhibitory concentration (IC_50_) was estimated by nonlinear regression using a four-parameter log-logistic model (LL.4) implemented in the drc package in R. Models were fitted separately for each nanoemulsion and fungal species using the individual mycelial growth inhibition values obtained from the four independent plates at each tested concentration. IC_50_ estimates are reported with their corresponding 95% confidence intervals.

### 4.7. Evaluation of the Effects of NEOEMS and NEOELC on Common Bean Seeds and Garlic Cloves

The effects of the nanoemulsions on common bean seed germination and early seedling development were evaluated on common bean (*Phaseolus vulgaris* L.) seeds by means of a standard germination test conducted according to the Rules for Seed Testing (RAS). The seeds were surface disinfected by immersion in a 2% (*v*/*v*) sodium hypochlorite solution for 2 min. For each nanoemulsion, three application rates (166.7, 333.3, and 500.0 mL per 100 kg of seeds), corresponding to 100, 200, and 300 μL applied to 60 g of seeds, respectively, were evaluated. The formulations were applied by pipetting the required volume directly onto the seeds placed in a sealed plastic bag, followed by manual mixing for 2 min to ensure uniform coating. The treated seeds were maintained in contact with the formulations for 30 min and subsequently dried on filter paper for an additional 30 min.

Following treatment, the seeds were distributed into four replicates of 50 seeds each. Distilled water and the blank nanoemulsion (without active compounds) were included as negative and formulation controls, respectively. The seeds were placed on Germitest^®^ paper moistened with distilled water at 2.5 times the dry weight of the paper, rolled, and incubated in a biochemical oxygen demand (BOD) incubator (Marconi MA415, Piracicaba, Brazil) at 25 °C. Germination was assessed on the ninth day after sowing according to the criteria established by the Rules for Seed Testing, and the results were expressed as the percentage of normal seedlings.

At the end of the assay, shoot and root lengths were measured in 10 randomly selected normal seedlings from each replicate. Fresh biomass was determined immediately after sampling, whereas dry biomass was obtained after drying the seedlings in a forced-air oven at 60 °C for 48 h. The experimental data were subjected to analysis of variance (ANOVA), followed by Tukey’s multiple comparison test at a significance level of 5% (*p* < 0.05) in R using RStudio version 2026.04.0+526 (Posit Software, PBC, Boston, MA, USA). The assumptions of normality and homogeneity of variance were verified, and data transformations were applied when necessary.

The effects of the nanoemulsions on garlic clove emergence and early growth were evaluated using an in vitro sprouting assay. Uniform-sized cloves were immersed for 30 min in distilled water, a blank nanoemulsion without active ingredient, iprodione, or NEOELC and NEOEMS at final active-phase concentrations of 250, 500, 750, 1000, 1500, and 2000 µg mL^−1^. After treatment, the cloves were removed from the solutions and placed individually in Gerbox^®^ plastic boxes (Winfield, KS, USA) containing three sheets of Germitest^®^ paper (São Paulo, Brazil) moistened with distilled water at an amount equivalent to 2.5 times the dry weight of the paper. The cloves were incubated in the dark at 23–25 °C, and the paper substrate was remoistened when necessary.

The experiment was conducted with four replicates of five garlic cloves per treatment. Emergence was defined as the appearance of roots and/or shoots and shoot, and root lengths were evaluated during the first seven days of development. The data were subjected to analysis of variance (ANOVA) after verification of residual normality using the Shapiro–Wilk test and homogeneity of variances using Levene’s test. When a significant effect was detected, means were compared using Tukey-adjusted multiple comparisons, adopting *p* < 0.05.

### 4.8. Evaluation of Garlic Under Field Conditions

A field experiment was conducted at the Federal University of Viçosa, Rio Paranaíba Campus (UFV-CRP), using garlic (*Allium sativum* L.) cultivar Ito. The experiment was arranged in a randomized complete block design (RCBD) with four blocks. Each experimental unit consisted of 30 garlic cloves, resulting in 120 cloves per treatment.

Three treatments were evaluated: NEOELC diluted to provide an *L*-carvone concentration of 1500 µg mL^−1^ in the treatment solution, NEOEMS diluted to provide an *M. spicata* essential oil concentration of 2000 µg mL^−1^ in the treatment solution, and iprodione as the conventional fungicide control. These concentrations refer to the respective active phases delivered through the nanoemulsions and were calculated from the active-phase content of each formulation. Before planting, the garlic cloves were immersed in the corresponding treatment solution for 30 min, removed from the solution, allowed to dry, and subsequently planted. The treatments were applied only once as a pre-planting clove treatment, and no additional applications were performed during the experiment.

Plant emergence was determined as the percentage of planted cloves that produced emerged shoots, and shoot length was measured as an indicator of early vegetative growth.

Data were analyzed according to the randomized complete block design. Treatment was considered a fixed effect and block a random effect. The assumptions of normality and homogeneity of variances were assessed before analysis. Data were subjected to analysis of variance (ANOVA), and when a significant treatment effect was detected, means were compared using Tukey’s multiple comparison test at *p* < 0.05.

### 4.9. Statistical Analysis

Physicochemical analyses of the nanoemulsions were performed in triplicate, and the results are presented as mean ± standard deviation (SD). Statistical procedures for the biological assays were selected according to the experimental design of each experiment.

Antifungal concentration–response data were analyzed separately for each phytopathogen and nanoemulsion using four-parameter log-logistic regression, as described in Section 4.6. IC_50_ values were obtained from the fitted models together with their corresponding 95% confidence intervals, whereas MIC values were determined experimentally as the lowest tested concentration resulting in complete inhibition of mycelial growth.

For the common bean seed-treatment assay, each combination of nanoemulsion formulation and application rate, together with the distilled water and blank nanoemulsion controls, was considered a treatment. Each response variable was subjected to analysis of variance (ANOVA), and treatment means were compared using Tukey’s multiple-comparison test at *p* < 0.05 when a significant treatment effect was detected.

For the in vitro garlic clove assay, formulation, concentration, and their interaction were considered in the statistical analysis. When appropriate, concentration effects were evaluated within each nanoemulsion, and NEOELC and NEOEMS were compared at equivalent concentrations using Tukey-adjusted multiple comparisons. Comparisons between nanoemulsion treatments and controls were conducted separately using Holm-adjusted multiple comparisons.

Data from the garlic experiment conducted under field conditions were analyzed according to a randomized complete block design, with treatment considered a fixed effect and block a random effect. When the treatment effect was significant, means were compared using Tukey’s multiple-comparison test at *p* < 0.05.

For ANOVA-based analyses, residual normality and homogeneity of variances were assessed using the Shapiro–Wilk and Levene tests, respectively. Data were transformed when necessary to meet model assumptions. Statistical analyses were performed in R using RStudio version 2026.04.0+526 (Posit Software, PBC, Boston, MA, USA).

## 5. Conclusions

NEOEMS and NEOELC formed oil-in-water nanoemulsions with nanometric droplet sizes and exhibited antifungal activity against the soil-borne phytopathogens *S. cepivora* and *S. sclerotiorum*. Among the evaluated formulations, NEOELC showed greater antifungal activity against *S. cepivora*, which may be related, at least in part, to the higher relative concentration of *L*-carvone in this formulation.

Neither formulation significantly impaired common bean seed germination or garlic clove emergence under the experimental conditions evaluated. However, reduced shoot growth was observed in garlic under field conditions, indicating that preservation of germination and emergence should not be interpreted as an overall absence of phytotoxic effects. As long-term field evaluations were not performed, further dose optimization and crop-safety assessment are required.

Both formulations retained their nanometric character during the evaluated storage period, although measurable changes in droplet size and PDI occurred. *L*-Carvone content was substantially preserved, with approximately 96.2% and 93.9% retention in NEOELC and NEOEMS, respectively, after 36 days. These findings indicate that colloidal and chemical stability should be considered independently when evaluating formulation performance.

Overall, the results support further investigation of NEOELC and NEOEMS as formulations for delivering natural antifungal compounds. Further studies should address long-term colloidal and chemical stability, crop compatibility, environmental persistence, efficacy under field conditions, and the mechanisms underlying their antifungal activity.

## Figures and Tables

**Table 1:** Chemical composition and tentative annotation of constituents detected in *Mentha spicata* essential oil by GC–MS.

Compound	Calculated AI	Reference AI (Adams) *	Similarity (%)	Relative Composition (%)
Limonene	1024	1024	95	11.21
*trans*-*p*-menthan-3-one	1148	1148	96	1.69
*cis*-*p*-menthan-3-one	1158	1158	93	0.26
NI **	1167			0.75
α-Terpineol	1186	1186	90	0.23
Dihydrocarveol	1190	1192	95	3.34
*cis*-Dihydrocarvone	1192	1191	94	2.57
*trans*-Dihydrocarvone	1200	1200	92	0.39
*trans*-Carveol	1215	1215	83	0.60
*cis*-Carveol	1228	1226	86	0.47
(3*Z*)-Hexenyl isopentanoate	1234	1232	84	0.19
*L*-Carvone	1242	1239	97	73.35
Menthyl acetate	1289	1294	95	0.42
Dihydrocarveyl acetate	1324	1326	93	1.64
β-Bourbonene	1376	1387	94	1.00
β-Elemene	1385	1389	95	0.42
β-Caryophyllene	1410	1417	95	1.47

* Reference arithmetic index reported by Adams [33]. ** NI, not identified.

**Table 2:** Analytical performance of the HPLC–DAD method used for the quantitative determination of *L*-carvone.

Parameter	Result
Linear range	0.05–1.00 mg mL^−1^
Calibration equation—standard in methanol	y = 4210.11x+ 19.17
Coefficient of determination (R^2^)	0.9994
Lack-of-fit	*p* = 0.104
Calibration equation—standard + placebo	y = 4221.26x+ 47.81
Coefficient of determination (R^2^), standard + placebo	0.9976
Lack-of-fit, standard + placebo	*p* = 0.206
Difference between slopes	0.26%
Comparison of slopes	*p* = 0.848
LOD	0.0293 mg mL^−1^
LOQ	0.0888 mg mL^−1^
Recovery, 80%	96.30 ± 1.07%
Recovery, 100%	101.05 ± 0.71%
Recovery, 120%	98.93 ± 3.51%
Overall recovery	98.76 ± 2.78%
Repeatability, RSD (*n* = 6)	3.34%

**Table 3:** Physicochemical properties of the NEOEMS and NEOELC nanoemulsions during 36 days of storage.

Parameter	NEOELC		NEOEMS	
	Day 1	Day 36	Day 1	Day 36
*L*-Carvone (mg mL^−1^)	42.86 ± 0.31	41.23 ± 2.32	27.68 ± 0.36	26.00 ± 0.10
Mean droplet size (nm)	80.8 ± 33.3	186.4 ± 21.7	119.6 ± 60.6	152.8 ± 11.8
Polydispersity index (PDI)	0.39 ± 0.09	0.20 ± 0.01	0.35 ± 0.01	0.27 ± 0.03
Zeta potential (mV)	−15.26 ± 1.34	−14.39 ± 4.17	−14.64 ± 2.52	−17.17 ± 2.69
Electrical conductivity (mS cm^−1^)	0.02 ± 0.00	0.12 ± 0.05	0.11 ± 0.15	0.10 ± 0.03
pH	7.30 ± 0.07	7.03 ± 0.12	6.54 ± 0.04	6.99 ± 0.11

**Table 4:** Antifungal activity of NEOELC and NEOEMS against *Stromatinia cepivora* and *Sclerotinia sclerotiorum*, expressed as IC_50_ and minimum inhibitory concentration (MIC). IC_50_ values were estimated using a four-parameter log-logistic regression model, whereas MIC was defined as the lowest tested concentration resulting in complete inhibition of mycelial growth.

Phytopathogen	Formulation	IC_50_ (µg mL^−1^), Estimate (95% CI)	MIC (µg mL^−1^)
*Stromatinia cepivora*	NEOELC	289.1 (255.6–322.6)	800
	NEOEMS	586.3 (304.3–868.2)	1600
*Sclerotinia sclerotiorum*	NEOELC	162.0 (133.6–196.4)	400
	NEOEMS	182.8 (142.5–234.8)	400

**Table 5:** Germination percentage (G), leaf fresh weight (LFW), leaf dry weight (LDW), root fresh weight (RFW), root dry weight (RDW), and root length (RL) of *Phaseolus vulgaris* seeds treated with NEOEMS, NEOELC, and the corresponding control treatments.

Treatment	G (%)	LFW (g)	LDW (g)	RFW (g)	RDW (g)	RL (cm)
NEOEMS 100 µL	97.0 a	9.0 a	1.8 ab	1.1 a	0.3 a	13.5 a
NEOEMS 200 µL	98.0 a	8.4 a	1.7 ab	1.3 a	0.2 a	13.7 a
NEOEMS 300 µL	96.0 a	7.9 a	1.4 b	1.5 a	0.2 a	13.6 a
NEOELC 100 µL	99.0 a	8.6 a	1.7 ab	1.3 a	0.2 a	13.6 a
NEOELC 200 µL	97.0 a	8.4 a	1.7 ab	1.2 a	0.2 a	13.6 a
NEOELC 300 µL	97.5 a	8.6 a	1.6 ab	1.6 a	0.2 a	14.1 a
Tween 80	99.0 a	8.6 a	1.9 a	1.4 a	0.3 a	14.1 a
Distilled water	99.0 a	8.1 a	1.6 ab	1.2 a	0.2 a	13.5 a

Values are presented as the mean of four replicates. Within each column, means followed by the same lowercase letter are not significantly different according to Tukey’s multiple comparison test (*p* < 0.05). The assumptions of normality and homogeneity of variances were verified using the Shapiro–Wilk and Levene’s tests, respectively.

**Table 6:** Shoot length and root length of *Allium sativum* cloves following treatment with different concentrations of NEOELC and NEOEMS.

**Concentration (µg mL^−1^)**	**NEOELC Shoot Length (cm)**	**NEOEMS Shoot Length (cm)**	**NEOELC Root Length (cm)**	**NEOEMS Root Length (cm)**
250	10.95 ± 1.07 cB	15.87 ± 1.61 bA	10.32 ± 1.65 aA	13.65 ± 0.85 aA
500	19.17 ± 2.10 bcA	23.82 ± 3.37 abA	13.45 ± 0.57 aA	15.20 ± 1.94 aA
750	22.61 ± 0.49 abA	24.96 ± 4.47 abA	9.95 ± 1.07 aB	16.52 ± 2.46 aA
1000	27.19 ± 4.76 abA	27.41 ± 3.50 abA	11.81 ± 1.73 aA	13.81 ± 0.37 aA
1500	30.70 ± 0.95 aA*	30.26 ± 2.62 aA*	8.82 ± 1.40 aA	10.03 ± 2.36 aA
2000	23.38 ± 1.42 abB	29.20 ± 1.82 abA	9.63 ± 1.31 aA	12.04 ± 1.30 aA

Values are expressed as the mean ± standard error (SE). Lowercase letters indicate comparisons among concentrations within each nanoemulsion, whereas uppercase letters indicate comparisons between NEOELC and NEOEMS at the same concentration for each variable. Means followed by the same letter are not significantly different according to Tukey’s multiple comparison test (*p* < 0.05). An asterisk (*) indicates a significant difference compared with the Tween 80 control after Holm’s correction (*p* < 0.05). No treatment differed significantly from either the distilled water or iprodione controls.

**Table 7:** Plant emergence and shoot length (SL) following treatment with NEOEMS and NEOELC compared with the iprodione control in a randomized complete block design (RCBD).

Treatment	Emergence (%)	Shoot Length (SL, cm)
Iprodione control	86.67 a	10.37 a
NEOEMS (2000 µg mL^−1^)	92.50 a	8.03 b
NEOELC (1500 µg mL^−1^)	85.83 a	7.46 b
CV (%)	7.31	9.37
Treatment (*p*-value)	0.3476	0.00499

Means followed by the same lowercase letter within a column are not significantly different according to Tukey’s multiple comparison test (*p* < 0.05).

## Data Availability

The original contributions presented in this study are included in the article. Further inquiries can be directed to the corresponding author.
